# Plasma biomarker profiles and the correlation with cognitive function across the clinical spectrum of Alzheimer’s disease

**DOI:** 10.1186/s13195-021-00864-x

**Published:** 2021-07-05

**Authors:** Zhenxu Xiao, Xue Wu, Wanqing Wu, Jingwei Yi, Xiaoniu Liang, Saineng Ding, Li Zheng, Jianfeng Luo, Hongchen Gu, Qianhua Zhao, Hong Xu, Ding Ding

**Affiliations:** 1 Institute of Neurology, Huashan Hospital, Fudan University, 12 Wulumuqi Zhong Rd, Shanghai, China; 2 National Clinical Research Center for Aging and Medicine, Huashan Hospital, Fudan University, Shanghai, China; 3 School of Biomedical Engineering, Shanghai Jiao Tong University, Shanghai, China; 4 Department of Biostatistics, School of Public Health, Fudan University, Shanghai, China; 5 Key Lab of Public Health Safety of the Ministry of Education, Fudan University, Shanghai, China

**Keywords:** Plasma p-tau181, Simoa, Cognitive domain, CDR, Alzheimer’s disease

## Abstract

**Background:**

Plasma biomarkers showed a promising value in the disease diagnosis and management of Alzheimer’s disease (AD). However, profiles of the biomarkers and the associations with cognition across a spectrum of cognitive stages have seldom been reported.

**Methods:**

We recruited 320 individuals with cognitive impairment and 131 cognitively normal participants from a memory clinic and a community cohort. Participants were classified into 6 groups based on their Clinical Dementia Rating (CDR) scores and clinical diagnosis, including AD, amnestic mild cognitive impairment (aMCI), and normal cognition (NC). A battery of neuropsychological tests was used to assess the global and domain-specific cognition. Plasma Aβ_1-40_, Aβ_1-42_, Aβ_1-42_/Aβ_1-40_, total tau (t-tau), neurofilament protein light chain (NfL), and phosphorylated tau at threonine 181 (p-tau181) were quantified using the single-molecule array (Simoa) platform.

**Results:**

All the plasma markers (Aβ_1-40_, Aβ_1-42_, Aβ_1-42_/Aβ_1-40_, t-tau, NfL, p-tau181) showed certain discrepancies among NC, aMCI, and AD groups. The p-tau181 level showed a continuous escalating trend as the CDR scores increased from 0 (NC group) to 3 (severe AD). Compared with other biomarkers, p-tau181 had correlations with broader cognitive domains, covering global cognition (*r* = −0.536, *P* < 0.0001), memory (*r* = −0.481, *P* < 0.0001), attention (*r* = −0.437, *P* < 0.0001), visuospatial function (*r* = −0.385, *P* < 0.0001), and language (*r* = −0.177, *P* = 0.0003). Among participants with CDR ≥ 1, higher p-tau181 was correlated with worse global cognition (*r* = −0.301, *P* < 0.001).

**Conclusions:**

Plasma p-tau181 had correlations with broader cognitive domains, suggesting its potential as a promising clinical-relevant blood-based biomarker.

**Supplementary Information:**

The online version contains supplementary material available at 10.1186/s13195-021-00864-x.

## Background

Alzheimer’s disease (AD), the most common cause of dementia, is characterized by the accumulation of the amyloid plaques and neuronal tangles in the brain [1]. Previous AD-relevant biomarkers could only be detected in the cerebrospinal fluid (CSF) or through positron emission tomography (PET) [1]. With the development of ultrasensitive immunoassays technique, detecting AD relevant biomarkers in blood samples became available [2]. Plasma biomarkers have a promising value in clinic usage due to the non-invasiveness, cost-effectiveness, and easy accessibility [3]. Following Aβ_1-42_, Aβ_1-40_, total tau (t-tau), neurofilament protein light chain (NfL), recently reported plasma phosphorylated tau at threonine 181 (p-tau181) showed better diagnostic performance and prognostic value in several cohort studies [3–9].

The cognitive performance is a pivotal indicator in AD management and efficacy evaluation. Previous studies found plasma Aβ_1-42_ [10], NfL [11, 12], and p-tau181 [3, 4, 13] were significantly different in participants with mild cognitive impairment (MCI) and AD compared with participants with normal cognition (NC). However, few studies depicted plasma biomarkers’ profiles based on the cognitive performance and compared their discrepancy in different stages of AD clinical syndrome. It would be valuable to reveal the alterations of plasma AD biomarkers from NC until the severe cognitive impairment stage since the cognitive manifestations are the most concerned issues for both clinicians, patients, and caregivers.

In addition, some studies investigated the relationships between plasma biomarkers and various cognitive domains [11, 14–22]. However, they only focused on individual markers, and the results were incomparable or inconsistent due to the diverse testing platforms and study designs. It is also worthwhile to observe which biomarker has the best correlation with the cognitive domains.

In this study, we aimed to depict the profile of plasma biomarkers, including Aβ_1-40_, Aβ_1-42_, Aβ_1-42_/Aβ_1-40_, t-tau, NfL, and p-tau181, among different stages of AD clinical syndrome. We also intended to explore the correlation between these biomarkers and the domain-specific cognitive functions.

## Methods

### Study participants

Participants with cognitive impairment were consecutively recruited from the memory clinic of the department of neurology, Huashan Hospital, Shanghai, China from November 2018 to September 2020. The inclusion criteria included the following: (1) consulted at the memory clinic due to memory complaints from herself (himself) or proxy; (2) able to cooperate with physical examinations and neuropsychological tests; (3) diagnosed with single-domain amnestic MCI (aMCI-s), multi-domain amnestic MCI (aMCI-m), or AD clinical syndrome; and (4) consent to the blood draw.

Participants in the Shanghai Aging Study (SAS) were eligible to be selected as the controls with NC. The SAS is a community-based longitudinal cohort in downtown Shanghai, China. The original purpose of SAS was to investigate the prevalence, incidence, and risk factors for dementia and MCI among older residents in an urban community. The detailed study design and recruitment procedure have been published elsewhere [23]. In this study, the participants were selected from the third wave of follow-up between Jun and Oct in 2020 if they were (1) 60 years or older, (2) able to cooperate with physical examinations and neuropsychological tests, (3) with normal cognition, and (4) consent to the blood draw.

### Demographics and assessment of covariables

The demographic and lifestyle characteristics were acquired from the participants and/or proxy through a questionnaire. The educational background was defined as the years of formal education. Participants who smoked daily within the past month were regarded as cigarette smoking, and alcohol consumption was defined as having at least one serving of alcohol weekly during the past year [24]. Hypertension and diabetes mellitus were confirmed by the medical records [23].

The Barthel index [25] and the Brody Activity of Daily Living (ADL) scale-16 [26] were used to elicit physical self-maintenance and instrumental activities of daily living, such as eating, preparing meals, using the telephone, handling money, and doing chores, for participants from the clinic and the community, respectively. Participants were considered functionally intact if the Barthel index score was over 60 [25], or the ADL score was over 16 [26]. Apolipoprotein E (APOE) genotype was assessed by the Taqman single-nucleotide polymorphism method using the blood or saliva samples collected from the participants [27]. The presence of at least one APOE ε4 allele was regarded as APOE ε4-positive.

### Neuropsychological tests

Comprehensive neuropsychological tests were administered by the certified psychometrists. All the tests were translated and adapted from western countries harmonized to Chinese culture and were validated in the Chinese population. Each participant from the memory clinic received a battery of neuropsychological tests, including (1) mini-mental status examination (MMSE) [28], (2) Montreal Cognitive Assessment-Basic (MoCA-B) [29–31], (3) Auditory Verbal Learning Test [32, 33], (4) Symbol Digit Modalities Test [34, 35], (5) Rey-Osterrieth Complex Figure test [35], (6) Boston Naming Test [36], and (7) Trail Making Test (TMT) [37]. For those who refused or were unable to complete the whole battery of tests, only MMSE and MoCA-B were administered. Because the MMSE is less sensitive for MCI detection [38], MoCA was used together with MMSE to discriminate dementia and MCI. As for the participants with NC, a battery of similar neuropsychological tests was administered due to the original study design of SAS [32]: (1) MMSE [28], (2) Auditory Verbal Learning Test [32, 33], (3) Conflicting Instructions Task (Go/No-Go Task) [32], (4) Stick Test [32], (5) Modified Common Objects Sorting Test [32], and (6) TMT [37].

In order to make the best use of the neuropsychological tests, we extracted raw scores from each test to evaluate five clinically relevant cognitive domains including Memory, Attention, Visuospatial function, Language, and Executive function (Supplementary Table 1). The percentage of the correct answer in each domain was calculated. Then, *Z* scores were further computerized to ensure the comparability between the participants from the two cohorts.

### Cognitive impairment severity and consensus diagnosis

All participants and their proxy were interviewed by two neurologists specialized in neurodegenerative diseases. The CDR, a semi-structured inventory, was used to assess the severity of cognitive impairment. It covered six cognitive, behavioral, and functional aspects, including memory, orientation, judgment and problem-solving, community affairs, home and hobby performance, and personal care. The neurologists scored on each aspect, taking into consideration information collected from both participants and proxy. The global CDR score was calculated automatically on biostat.wustl.edu/~adrc/cdrpgm/index.html by inputting in each CDR box score, based on the Washington University CDR-assignment algorithm with a 0–3 scale index [39, 40].

Neurologists and neuropsychologists reached a consensus diagnosis of the cognitive function of each participant. The presence or absence of dementia was defined using the DSM-IV criteria [41]: (1) memory impairment; (2) at least one of the following cognitive impairments: aphasia, apraxia, agnosia, and executive dysfunction; (3) cognitive deficit results in significant impairment of social or professional functioning; (4) cognitive function declines slowly; and (5) exclude other potential disorders that contribute to cognitive decline. Probable AD was diagnosed according to the NINCDS-ADRDA criteria [42]: (1) dementia established by MMSE and confirmed by neuropsychological tests, (2) deficits in two or more areas of cognition, (3) progressive worsening of memory and other cognitive functions, (4) no disturbance of consciousness, and (5) the laboratory tests (including, thyroid function, syphilis tests, level of vitamin B12 and folate, and other related blood tests) and magnetic resonance imaging (MRI)/computerized tomography (CT) was performed to rule out other systemic disorders or other brain diseases that would cause cognitive decline. Participants who met the criterion of probable AD were regarded as having AD clinical syndrome. The diagnosis of MCI was based on Petersen’s criteria [43]: (1) cognitive complaint by the subject, informant, nurse, or physician, with CDR = 0.5; (2) objective impairment in at least 1 cognitive domain; (3) essentially normal functional activities (determined from the CDR and the ADL score); (4) absence of dementia; and (5) the laboratory tests and MRI/CT was performed to rule out other systemic disorders or other brain diseases that would cause cognitive decline [44]. Because aMCI is more likely to progress to AD [45], we only included participants with 2 types of aMCI: (1) aMCI-s, memory impairment was required with no deficit in other domains, and (2) aMCI-m, memory impairment plus at least 1 additional deficit in another domain. NC participants had no memory complaint and have been confirmed cognitively intact through detailed neuropsychological assessment.

In this study, the continuum of AD clinical syndrome was described based on the combination of CDR and clinical diagnosis: NC (CDR = 0), aMCI-s (CDR = 0.5), aMCI-m (CDR = 0.5), mild AD (CDR = 1), moderate AD (CDR = 2), and severe AD (CDR = 3) [39, 40].

### Plasma biomarker measurement

Blood samples were collected in ethylene diamine tetraacetic acid (EDTA) plasma tubes and centrifuged (1000 rpm, 4°C) for 15 min. After the centrifugation, plasma was transferred into 1.5ml Eppendorf tubes and stored at −80 °C refrigerators.

Plasma Aβ_1-40_, Aβ_1-42_, t-tau, NfL, and p-tau181 were quantified using an ultra-sensitive single-molecule array (Simoa) (Quanterix, MA, USA) on the automated Simoa HD-X platform per the manufacturer’s instruction. The multiplex Neurology 3-Plex A kits (Cat. No. 101995), NF-light assay (Cat. No. 103186), and p-tau181 Assay Kit V2 (Cat. No.103714) were purchased from Quanterix and used accordingly. Technicians who performed the assay were blinded to the clinical data.

### Statistical analyses

Mean and standard deviation (SD) were used to describe normally distributed continuous variables, while the median and quartile 1 (Q1) to quartile 3 (Q3) were used to describe the skewed distributed continuous variables. For categorical variables, number (n) and frequencies (%) were employed. One-way analyses of variance (ANOVA) and Kruskal-Wallis tests were used for comparing continuous variables, and Pearson chi-square and Fisher exact tests were used to compare the categorical variables. For the comparisons among multi-groups, ANOVA and post hoc Tukey multiple comparison tests were used for variables with equal variance, while Welch and post hoc Games-Howell tests were used for variables with unequal variance.

Boxplots and points were used to present the distributions of original values of six plasma biomarkers. Levene’s tests were used for testing the homogeneity of variances in six groups with different cognition statuses. Associations between domain-specific *Z* scores and log-transformed plasma biomarker indexes (due to non-normal distributions [3]) were examined using partial correlation analyses with the adjustment for age, gender, and education year. The heatmap matrix was implemented to visualize the adjusted partial correlation coefficients *r* in all participants. Positive correlations (*r* > 0) were exhibited in red, and negative correlations (*r* < 0) were exhibited in blue in the heatmap figure. The same correlation analyses were applied to participants with CDR = 0, CDR = 0.5, and CDR ≥ 1, respectively. For partial correlation analysis, we used the Bonferroni method for multiple corrections [46]. Since global cognition and memory are the two most important cognition domains for AD clinical syndrome, scatter plots were used to illustrate the correlations between MMSE & memory and plasma biomarkers.

Two-sided *P* < 0.05 was considered statistically significant, except for a specific explanation of multiple corrections. The degree of freedoms (df) was presented in correlation analysis. Data were analyzed using IBM SPSS Statistics for Windows, version 25.0 (IBMCorp., Armonk, N.Y., USA) [47] and R (version 4.0.2) [48]. Box plots were produced using the package ggplot2 in R. The heatmap was visualized by GraphPad Prism version 8.0.0 for Windows, GraphPad Software, San Diego, California, USA (www.graphpad.com).

## Results

### Characteristics of the study participants

We recruited 451 participants, including 320 participants from the memory clinic cohort and 131 NC participants from the community cohort. The characteristics of study participants were shown in Table 1. Significant difference was found in gender (*P* = 0.011), age (*P* < 0.001), education years (*P* < 0.001), and APOE ε4 allele (*P* < 0.001) among six cognitive performance groups. The most severe cognitive impairment group (CDR = 3) had the highest proportion of women (68.4%), the lowest mean age (mean = 62.3), the shortest education years (mean = 5.6), and the largest proportion of positive APOE ε4 allele (57.9 %). We did not find a significant discrepancy in smoking, alcohol consumption, hypertension, and diabetes mellitus among the six groups. All neuropsychological test scores were significantly different among six groups (all *P* < 0.001).

**Table 1:** Demographic characteristics and the neuropsychological assessments among study participants

	Total	Clinical diagnosis						
		NC	aMCI-s	aMCI-m	Mild AD	Moderate AD	Severe AD	
		CDR = 0	CDR = 0.5	CDR = 0.5	CDR = 1	CDR = 2	CDR = 3	
*\Demographic characteristics*	(*N* = 451)	(*N* = 131)	(*N* = 39)	(*N* = 113)	(*N* = 67)	(*N* = 63)	(*N* = 38)	*P value*
Gender, female, n (%)	260 (57.6)	82 (62.6)	14 (35.9)	69 (61.1)	31 (46.3)	38 (60.3)	26 (68.4)	0.011
Age, y, mean (SD)	68.6 (8.8)	69.2 (7.1)	69.6 (9.3)	70.0 (8.0)	67.7 (9.7)	69.1 (10.8)	62.3 (8.2)	<0.001
Education years, mean (SD)	10.3 (4.1)	11.8 (2.9)	13.6 (3.6)	10.2 (3.6)	10.3 (3.6)	8.4 (4.6)	5.6 (4.0)	<0.001
Smoking, n (%)	79 (17.5)	17 (13.0)	5 (12.8)	23 (20.4)	15 (22.4)	12 (19.0)	7 (18.4)	0.529
Alcohol consumption, n (%)	77 (17.1)	19 (14.5)	4 (10.3)	21 (18.6)	18 (26.9)	12 (19.0)	3 (7.9)	0.139
Hypertension, n (%)	168 (37.3)	53 (40.5)	17 (43.6)	44 (38.9)	23 (34.3)	22 (34.9)	9 (23.7)	0.461
Diabetes mellitus, n (%)	68 (15.1)	19 (14.5)	7 (17.9)	19 (16.8)	8 (11.9)	10 (15.9)	5 (13.2)	0.933
APOE ε4-positive, n (%)	161 (35.7)	13 (9.9)	12 (30.8)	48 (42.5)	30 (44.8)	36 (57.1)	22 (57.9)	<0.001
*Neuropsychological tests*								
MMSE score, mean (SD)	23.3 (6.7)	29.0 (1.3)	27.7 (1.7)	25.8 (1.9)	21.9 (1.4)	15.7 (2.3)	7.4 (2.6)	<0.001
Memory^a^, median [Q1, Q3]	−0.07 [−1.18, 0.72]	0.87 [0.56, 1.50]	0.16 [−0.23, 0.72]	−0.23 [−0.71, 0.72]	−0.71 [−1.18, −0.23]	−1.18 [−1.18, −0.82]	−1.18 [−1.18, −1.18]	<0.001
Execution function^a^, median [Q1, Q3]	0.43 [−1.25, 0.43]	0.43 [0.43, 0.43]	0.43 [0.43, 1.49]	−0.41 [−1.25, 1.28]	0.43 [−1.25, 0.43]	−1.25 [−1.25, −0.41]	−1.25 [−1.25, −1.25]	<0.001
Attention^a^, median [Q1, Q3]	0.43 [−0.39, 0.83]	0.83 [0.67, 0.83]	0.69 [0.40, 0.83]	0.43 [−0.21, 0.83]	−0.36 [−0.78, 0.43]	−1.46 [−1.99, −0.39]	−2.01 [−2.42, −2.01]	<0.001
Language^a^, median [Q1, Q3]	0.58 [−0.27, 0.72]	0.72 [0.58, 0.72]	0.58 [0.16, 0.72]	−0.27 [−0.66, 0.72]	−0.27 [−1.16, 0.72]	−0.27 [−1.26, 0.72]	−1.26 [−2.25, −0.27]	<0.001
Visuospatial function^a^, median [Q1, Q3]	0.31 [−0.63, 0.93]	0.93 [0.62, 0.93]	0.76 [0.31, 0.93]	0.15 [−0.32, 0.62]	−0.63 [−1.25, −0.005]	−1.25 [−1.87, −0.32]	−1.87 [−2.18, −1.02]	<0.001

Note: ^a^Z score transformed. *NC*, normal cognition; *aMCI-s*, amnestic mild cognitive impairment-single domain; *aMCI-m*, amnestic mild cognitive impairment-multiple domains; *AD*, Alzheimer’s disease clinical syndrome; *CDR*, Clinical Dementia Rating Scale; *SD*, standard deviation; *Q1*, quartile 1; *Q3*, quartile 3; *APOE*, apolipoprotein E; *MMSE*, mini-mental state examination

### Plasma biomarkers across groups with different cognitive performances

**Fig. 1:**
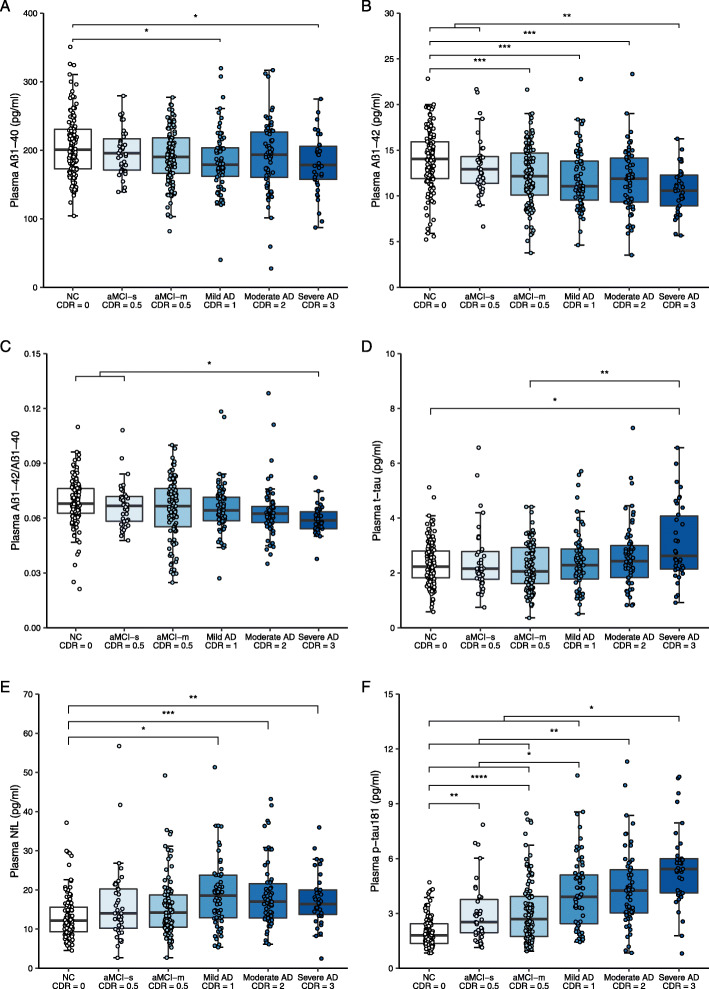
Plasma biomarkers in participants with different clinical cognitive status. Note: The ANOVA and the post hoc Tukey test were used for comparison of plasma Aβ_1-40_ & Aβ_1-42_ in six groups, while Welch test and the post hoc Games-Howell test were used to compare the plasma Aβ_1-42_/Aβ_1-40_, t-tau, NfL, and p-tau181 among six groups. Six extreme values were not shown in panel E, but they were included in the statistical analyses. The multiple corrected *P* values were presented with asterisks: *P < 0.05, **P < 0.01, ***P < 0.001, and ****P < 0.0001. NC, normal cognition; aMCI-s, amnestic mild cognitive impairment-single domain; aMCI-m, amnestic mild cognitive impairment-multiple domains; AD, Alzheimer’s disease clinical syndrome; CDR, Clinical Dementia Rating Scale; Aβ, amyloid-beta protein; t-tau, total tau; NfL, neurofilament protein light chain; p-tau181, tau phosphorylated at threonine 181

As shown in Fig. 1, plasma Aβ_1-40_, Aβ_1-42_, and Aβ_1-42_/Aβ_1-40_ ratio showed a descending trend, while plasma t-tau, NfL, and p-tau181 exhibited an increasing trend across groups with the increasing CDR scores in general. With regard to Aβ_1-40_, Aβ_1-42,_ and NfL, we found significant differences between participants with NC (CDR = 0) and AD (CDR ≥ 1) (Fig. 1A, B, and E). Aβ_1-42_/Aβ_1-40_ and t-tau showed differences only between participants with NC (CDR = 0) and severe AD (CDR = 3) (Fig. 1C and D). There was no significant discrepancy of Aβ_1-40_, Aβ_1-42_, Aβ_1-42_/Aβ_1-40_, t-tau, or NfL among participants with different severity levels of AD (CDR = 1, 2, or 3). P-tau181 gradually increased among different cognitive stages of AD clinical syndrome, with the lowest concentration in NC participants (CDR = 0), an increase in participants with aMCI (CDR = 0.5), and the highest concentration in participants with AD (CDR ≥ 1) (Fig. 1F). Specifically, participants with severe AD (CDR = 3) had significantly higher p-tau181 than those with mild AD (CDR =1).

When we compared the difference of plasma biomarkers between CDR = 0.5 and CDR >=1, it turned out that plasma Aβ_1-42_ (*P* = 0.025), t-tau (*P* = 0.010), and p-tau181 (*P* < 0.001) showed significant difference (not shown in Fig. 1).

### Correlation between plasma biomarkers and domain-specific cognition

**Fig. 2:**
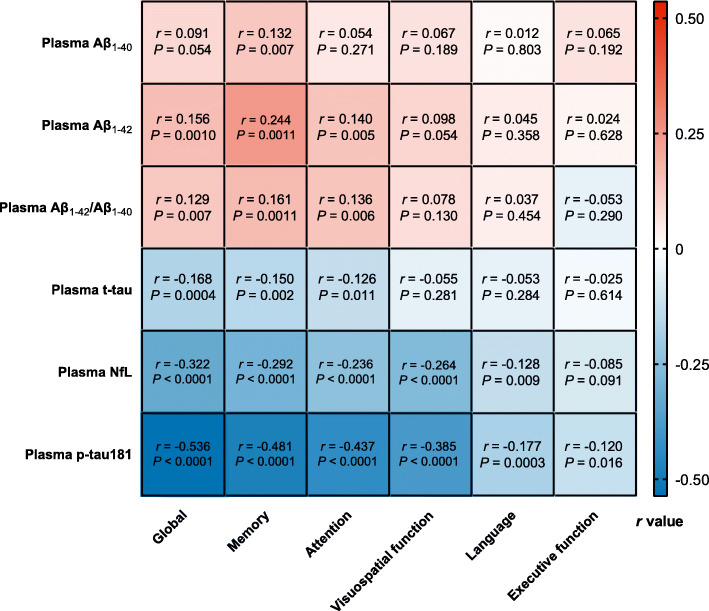
Correlations between plasma biomarkers and cognitive domains. Note: The plasma biomarkers concentrations were log transformed. The partial correlation coefficients (*r*) were adjusted for age, gender, and education year. P < 0.0014 was considered statistically significant after using multiple comparisons by Bonferroni correction. Aβ, amyloid-beta protein; t-tau, total tau; NfL, neurofilament protein light chain; p-tau181, tau phosphorylated at threonine 181

**Fig. 3:**
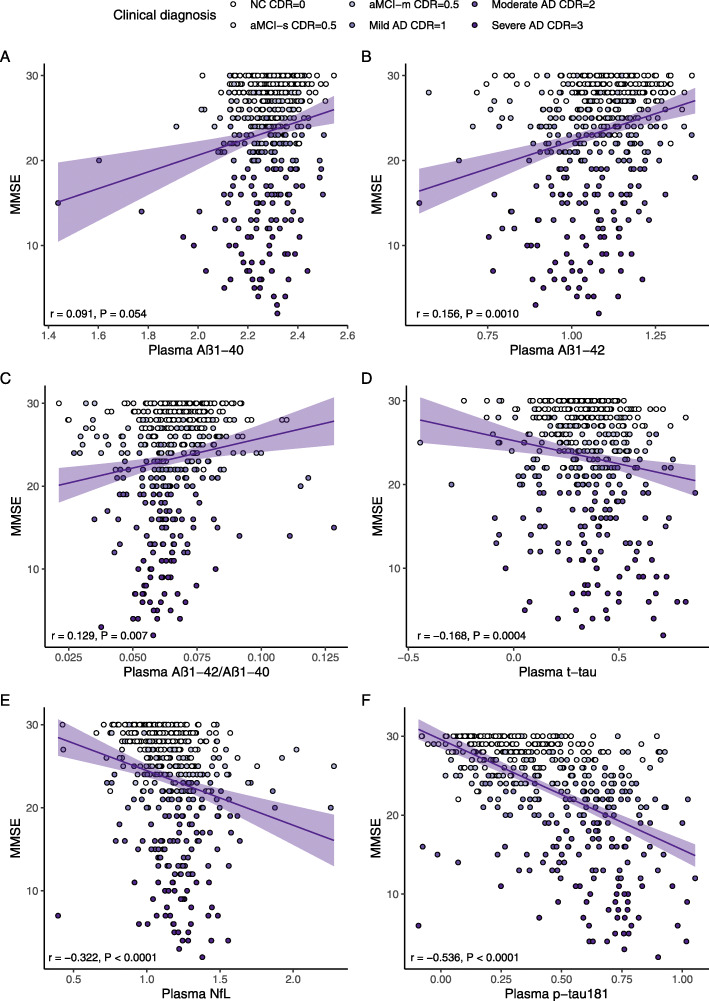
Scatter plots of MMSE and plasma biomarkers. Note: The plasma biomarkers concentrations were log-transformed. The partial correlation coefficients (*r*) were adjusted for age, gender, and education year. P < 0.0014 was considered statistically significant after using multiple comparisons by Bonferroni correction. The purple area represented the 95% confidence interval. MMSE, mini-mental state examination; Aβ, amyloid-beta protein; t-tau, total tau; NfL, neurofilament protein light chain; p-tau181, tau phosphorylated at threonine 181

**Fig. 4:**
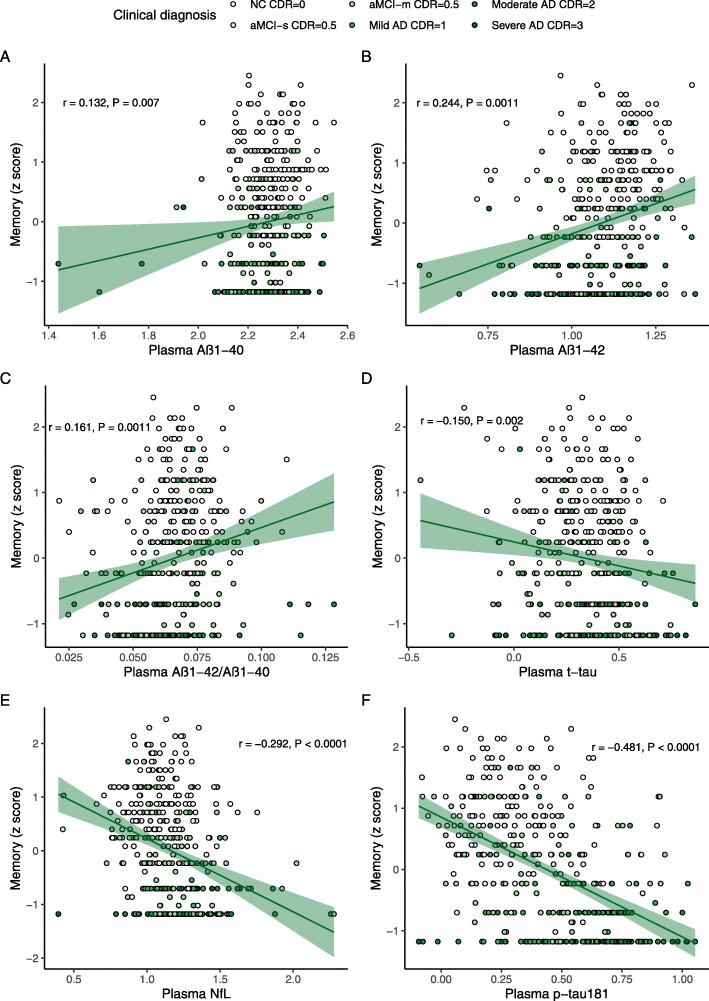
Scatter plots of Memory and plasma biomarkers. Note: The plasma biomarkers concentrations were log-transformed. The partial correlation coefficients (*r*) were adjusted for age, gender, and education year. *P* < 0.0014 was considered statistically significant after using multiple comparisons by Bonferroni correction. The green area represented the 95% confidence interval. MMSE, mini-mental state examination; Aβ, amyloid-beta protein; t-tau, total tau; NfL, neurofilament protein light chain; p-tau181, tau phosphorylated at threonine 181

Figure 2 showed the partial correlation matrix between six plasma biomarkers and six domain-specific cognitions after adjusting age, gender, and education years. Through Bonferroni correction, Aβ_1-42_ showed a positive correlation with MMSE (*r* = 0.156, df = 444, *P* = 0.0010) and memory (*r* = 0.244, df = 408, *P* < 0.0011). Aβ_1-42_/Aβ_1-40_ was only positively correlated with memory (*r* = 0.161, df = 407, *P* = 0.0011). T-tau had an inverse correlation with MMSE (*r* = -0.168, df = 444, *P* = 0.0004). Higher NFL was correlated with worse MMSE (*r* = -0.322, df = 441, *P* < 0.0001), memory (*r* = -0.292, df = 405, *P* < 0.0001), attention (*r* = -0.236, df = 406, *P* < 0.0001), and visuospatial function (*r* = −0.264, df = 378, *P* < 0.0001). P-tau181 showed a stronger negative correlation with MMSE (*r* = −0.536, df = 441, *P* < 0.0001), memory (*r* = −0.481, df = 406, *P* < 0.0001), attention (*r* = −0.437, df = 407, *P* < 0.0001), visuospatial function (*r* = −0.385, df = 379, *P* < 0.0001), and language (*r* = −0.177, df = 406, *P* = 0.0003). Figures 3 and 4 demonstrated the scatter plots of the correlations between MMSE & memory and six plasma biomarkers, respectively.

When the participants were divided into three stages according to the CDR score, only higher p-tau181 was correlated with worse MMSE in participants with CDR ≥ 1 after Bonferroni correction (*r* = −0.301, df = 161, *P* < 0.001) (Supplementary Table 2).

## Discussion

The present study demonstrated that plasma Aβ_1-40_, Aβ_1-42_, and Aβ_1-42_/Aβ_1-40_ had a decreasing trend, while plasma t-tau, NfL, and p-tau181 escalated along the different stages of cognitive impairment as the CDR score increased from 0 to 3. P-tau181 was the best plasma indicator of clinical cognitive performance and had correlations with broader cognitive domains than the other five biomarkers. To our knowledge, it was the first study to exhibit the distribution of plasma p-Tau 181 using the Simoa HD-X platform in Chinese older individuals with different stages of AD clinical syndrome.

Pathology is pivotal in the diagnosis of AD. However, cognitive performance, including various cognitive domains, played a more significant role in patient management and efficacy evaluation. Cognitive manifestations are closely related to one’s daily function and quality of life for both patients and caregivers. Thus, we focused on individual’s performance and classified the participants into six groups according to CDR levels and clinical cognitive diagnosis. One previous study found plasma p-tau181 increased at preclinical AD and further increased at the MCI and dementia stages [3]. Another group verified that plasma p-tau181 gradually increased from the Aβ-negative cognitively unimpaired older adults, through the Aβ-positive cognitively unimpaired and MCI groups, to the highest concentrations in Aβ-positive MCI and AD groups [4]. Our results testified their findings from the clinical perspective and further indicated that p-tau181 was a symptom-related plasma biomarker. Although some markers showed a significant escalating or descending trend along the AD spectrum, plasma p-tau181 is the only one that had a significant discrepancy in the later stage of AD clinical syndrome with overt dementia symptoms. This means plasma p-tau181 may keep increasing along with the deterioration of cognitive function, till the severe stage of AD. Previous studies showed that plasma p-tau181 was correlated with CSF p-tau181 in AD patients, suggesting that plasma p-tau181 was originated from the central nervous system [3, 4]. Peripheral p-tau reflects the phosphorylation of the tau protein, which eventually leads to the neurofibrillary tangles in the brain [8]. Therefore, the continuous increment of the plasma p-tau across different stages of AD indicated the ongoing tau-related pathologic change in the brain, even in the moderate to severe stage of the disease. This may potentially guide the development of disease-modifying therapy for the advanced-stage AD patients in the future.

Previously, one cohort study demonstrated that plasma p-tau181 was correlated with the MMSE score [4]. However, the relationships between plasma p-tau181 and different cognitive domains have not been reported. Our study revealed a strong correlation between plasma p-tau181 and various cognitive domains. The correlation between p-tau181 and MMSE was only observed in participants with dementia symptoms. This may reflect the underlying mechanism that plasma p-tau181 is regulated differently by the disease staging, namely the Alzheimer’s pathological status in the brain. Another possibility is that the narrow range of the testing scales, as well as the “ceiling effects” or “floor effects,” might also weaken the correlation between plasma biomarkers and cognition. However, this might not be the main reason, since when analyzing with the more sensitive tests assessing memory and attention, the correlations were still absent in the subgroup with CDR = 0.

The NfL was an age-related biomarker [49]. Lin et al. found that higher plasma NfL levels correlated with lower MMSE scores [15], without adjustment for age. Another study by Chatterjee et. al. showed that plasma NfL was inversely correlated with working memory, executive function, and the global composite score after considering age [14]. Two other studies found plasma NfL associated with all cognitive domains after adjusting potential confounders including age [21, 22]. However, using the same Simoa detecting method, a Chinese group did not find any correlation of NfL with episodic memory, information processing speed, executive function, or visuospatial function after adjusting for age, gender, and education. In our results, the plasma NfL had a significant correlation after adjustment for age and other covariates, not only with global cognition, but also with the other three cognitive domains. This suggested that, although NfL was regarded as a non-specific biomarker for neurological diseases [12], it still has value in the monitoring of AD cognitive deterioration. Nevertheless, most correlations could not be observed when we classified participants into three sub-groups according to CDR levels of 0, 0.5, and ≥1. The paradoxical results may be attributed to the diverse pathophysiological conditions in different cognitive stages [49].

The traditional amyloid cascade theory emphasized that Aβ as an initial factor that triggers the following Tau pathology [1]. Our study found a relatively weaker correlation between the plasma amyloid biomarkers (Aβ_1-42_, Aβ_1-42_/Aβ_1-40_) and the cognition, only with MMSE and memory. Previous CSF and PET studies [50, 51] indicated that the amyloid-related biomarkers reflected the earliest pathological change but tended to reach a plateau as the disease progressed to the dementia stage. Since the plasma Aβ was supposed to display the central nervous change [52], blood Aβ level may also be saturated in the symptomatic individuals. However, in the CDR subgroups, the trend is ambiguous and inconsistent, probably because of the small sample and minor effect size. Further large-scale longitudinal studies need to be conducted to demonstrate the dynamic Aβ alteration along the spectrum of AD. A previous experimental study showed that neurons exposed to Aβ had increased synthesis and secretion of tau [53]. These neurons may eventually degenerate and develop tangle pathology, which may also drive the increase of p-tau in the blood. Therefore, regarding AD as a lengthy, continuous disease course, plasma p-tau181, being more sensitive and clinical-relevant, might be superior to the amyloid-related markers.

### Limitations

There are several limitations to our study. Firstly, the biomarkers in this study were only detected once without longitudinal measurements. However, we separated the participants into six groups according to CDR scores and cognitive diagnosis to simulate the spectrum of AD clinical syndrome. Future prospective studies are needed to verify our findings. Secondly, the participants in our studies were from two different resources with unavoidable imparity. However, there was no significant difference in some dementia-related risk factors, such as smoking, alcohol consumption, hypertension, and diabetes mellitus (Table 1). Age, gender, and education year were adjusted in the multivariate statistical models. Thirdly, some neuropsychological tests might have “ceiling effects” (e.g., Go/No-Go Task) or “floor effects” (e.g., Auditory Verbal Learning Test), which may conceal the relationship between the biomarkers and the cognitive domains. Tests assessing similar cognitive domains have unavoidable learning effects [54, 55] and intrusion errors [56], which may lead to the unstable evaluation of the cognitive performance of the study participants. In our study, 38.6% of the intrusion rate of the memory tests (Supplementary Table 3) may impact the correlation estimations between the biomarkers and the cognitive domains. Fourthly, not all the participants were able to accomplish the extensive testing. About 90% of the participants accomplished tests for domains of memory, executive function, attention, and language, while 86% accomplished tests for the domain of the visuospatial function. Particularly in participants with severe AD, the response rates were even lower, due to their lack of ability to accomplish all the tests. When we repeated the analyses in participants that were able to accomplish all the tests (N = 374), the most correlations still hold, including associations between p-tau181, NfL and global cognition, memory, attention, and visuospatial function; between t-tau and global cognition; and between Aβ42, Aβ42/Aβ40, and memory (Supplementary Table 4). Although the correlation between plasma A42 and global cognition, language and p-tau181 could not pass the Bonferroni correction, and the *P* values were still < 0.01. Considering the Bonferroni correction as a conservative test, the results and conclusions could still be seen as solid. Fifthly, since AD was regarded as a continuum, the stage-partitioned correlations should be interpreted with caution due to the restricted range of cognitive test scores within each partition of disease severity. Lastly, we diagnosed AD based on the clinical standard rather than pathological evidence of CSF or amyloid/tau PET. Lacking a golden standard impeded us from the classifying of “ATN” framework [57] or receiver operating characteristic analysis.

## Conclusions

In conclusion, we found plasma p-tau181 increased in the AD clinical syndrome. Plasma p-tau181 had correlations with broader cognitive domains than other biomarkers. Our study suggests that the plasma p-tau181 may be a promising, clinical-relevant blood-based biomarker. Longitudinal studies are needed to verify these findings and provide more evidence for the association between plasma p-tau181 and cognitive manifestations.

## Supplementary Information

**Additional file 1: Supplementary Table 1.** Domain-specific cognition extracted from neuropsychological tests in the current study.

**Additional file 2: Supplementary Table 2.** Correlations between plasma biomarkers and global & domain-specific cognition in participants with different AD stages

**Additional file 3: Supplementary Table 3.** Intrusion of the Memory tests.

**Additional file 4: Supplementary Table 4.** Correlations between plasma biomarkers and cognitive domains in participants that were able to accomplish all tests (*N* = 374).

## Data Availability

The datasets used and/or analyzed during the current study are available from the corresponding author on reasonable request.

## Abbreviations

AD: Alzheimer’s disease
aMCI: Amnestic mild cognitive impairment
aMCI-m: Amnestic mild cognitive impairment multi-domains
aMCI-s: Amnestic mild cognitive impairment single-domain
ANOVA: Analysis of variance
APOE: Apolipoprotein E
CDR: Clinical Dementia Rating
CSF: Cerebrospinal fluid
EDTA: Ethylene diamine tetraacetic acid
MCI: Mild cognitive impairment
MMSE: Mini-mental state examination
MoCA-B: Montreal Cognitive Assessment-Basic
NC: Normal cognition
NfL: Neurofilament protein light chain
PET: Positron emission tomography
p-tau181: Phosphorylated tau 181
SAS: Shanghai Aging Study
SD: Standard deviation
Simoa: Single-molecule array
TMT: Trail Making Test
t-tau: Total tau

## References

[1] Scheltens P, Blennow K, Breteler MMB, de Strooper B, Frisoni GB, Salloway S (2016). Alzheimer’s disease. Lancet.

[2] Blennow K, Zetterberg H (2018). Biomarkers for Alzheimer’s disease: current status and prospects for the future. J Intern Med.

[3] Janelidze S, Mattsson N, Palmqvist S, Smith R, Beach TG, Serrano GE, Chai X, Proctor NK, Eichenlaub U, Zetterberg H, Blennow K, Reiman EM, Stomrud E, Dage JL, Hansson O (2020). Plasma P-tau181 in Alzheimer’s disease: relationship to other biomarkers, differential diagnosis, neuropathology and longitudinal progression to Alzheimer’s dementia. Nat Med.

[4] Karikari TK, Pascoal TA, Ashton NJ, Janelidze S, Benedet AL, Rodriguez JL, Chamoun M, Savard M, Kang MS, Therriault J, Schöll M, Massarweh G, Soucy JP, Höglund K, Brinkmalm G, Mattsson N, Palmqvist S, Gauthier S, Stomrud E, Zetterberg H, Hansson O, Rosa-Neto P, Blennow K (2020). Blood phosphorylated tau 181 as a biomarker for Alzheimer’s disease: a diagnostic performance and prediction modelling study using data from four prospective cohorts. Lancet Neurol.

[5] Lantero Rodriguez J, Karikari TK, Suarez-Calvet M, Troakes C, King A, Emersic A (2020). Plasma p-tau181 accurately predicts Alzheimer’s disease pathology at least 8 years prior to post-mortem and improves the clinical characterisation of cognitive decline. Acta Neuropathol.

[6] Cullen NC, Leuzy A, Palmqvist S, Janelidze S, Stomrud E, Pesini P (2020). Individualized prognosis of cognitive decline and dementia in mild cognitive impairment based on plasma biomarker combinations. Nat Aging.

[7] Park JC, Han SH, Yi D, Byun MS, Lee JH, Jang S, Ko K, Jeon SY, Lee YS, Kim YK, Lee DY, Mook-Jung I (2019). Plasma tau/amyloid-beta1-42 ratio predicts brain tau deposition and neurodegeneration in Alzheimer’s disease. Brain..

[8] Mielke MM, Hagen CE, Xu J, Chai X, Vemuri P, Lowe VJ, Airey DC, Knopman DS, Roberts RO, Machulda MM, Jack CR, Petersen RC, Dage JL (2018). Plasma phospho-tau181 increases with Alzheimer’s disease clinical severity and is associated with tau- and amyloid-positron emission tomography. Alzheimers Dement.

[9] Barthélemy NR, Horie K, Sato C, Bateman RJ (2020). Blood plasma phosphorylated-tau isoforms track CNS change in Alzheimer’s disease. J Exp Med.

[10] Albani D, Marizzoni M, Ferrari C, Fusco F, Boeri L, Raimondi I (2019). Plasma Aβ42 as a biomarker of prodromal Alzheimer’s disease progression in patients with amnestic mild cognitive impairment: evidence from the PharmaCog/E-ADNI study. J Alzheimers Dis.

[11] Zhou W, Zhang J, Ye F, Xu G, Su H, Su Y, Zhang X, Alzheimer’s Disease Neuroimaging Initiative (2017). Plasma neurofilament light chain levels in Alzheimer’s disease. Neurosci Lett.

[12] Mattsson N, Andreasson U, Zetterberg H, Blennow K (2017). Association of plasma neurofilament light with neurodegeneration in patients with Alzheimer disease. JAMA Neurol.

[13] Karikari TK, Benedet AL, Ashton NJ, Lantero Rodriguez J, Snellman A, Suarez-Calvet M (2021). Diagnostic performance and prediction of clinical progression of plasma phospho-tau181 in the Alzheimer’s disease neuroimaging initiative. Mol Psychiatry.

[14] Chatterjee P, Goozee K, Sohrabi HR, Shen K, Shah T, Asih PR, Dave P, ManYan C, Taddei K, Chung R, Zetterberg H, Blennow K, Martins RN (2018). Association of plasma neurofilament light chain with neocortical amyloid-beta load and cognitive performance in cognitively normal elderly participants. J Alzheimers Dis.

[15] Lin YS, Lee WJ, Wang SJ, Fuh JL (2018). Levels of plasma neurofilament light chain and cognitive function in patients with Alzheimer or Parkinson disease. Sci Rep.

[16] Shi Y, Lu X, Zhang L, Shu H, Gu L, Wang Z, Gao L, Zhu J, Zhang H, Zhou D, Zhang Z (2019). Potential value of plasma amyloid-beta, total tau, and neurofilament light for identification of early Alzheimer’s disease. ACS Chem Neurosci.

[17] Lee JJ, Choi Y, Chung S, Yoon DH, Choi SH, Kang SM, Seo D, Park KI (2020). Association of plasma oligomerized beta amyloid with neurocognitive battery using Korean version of consortium to establish a registry for Alzheimer’s disease in health screening population. Diagnostics (Basel).

[18] Lim YY, Maruff P, Kaneko N, Doecke J, Fowler C, Villemagne VL, Kato T, Rowe CC, Arahata Y, Iwamoto S, Ito K, Tanaka K, Yanagisawa K, Masters CL, Nakamura A (2020). Plasma amyloid-beta biomarker associated with cognitive decline in preclinical Alzheimer’s disease. J Alzheimers Dis.

[19] Tsai CL, Liang CS, Lee JT, Su MW, Lin CC, Chu HT, Tsai CK, Lin GY, Lin YK, Yang FC (2019). Associations between plasma biomarkers and cognition in patients with Alzheimer’s disease and amnestic mild cognitive impairment: a cross-sectional and longitudinal study. J Clin Med.

[20] Sugarman MA, Zetterberg H, Blennow K, Tripodis Y, McKee AC, Stein TD (2020). A longitudinal examination of plasma neurofilament light and total tau for the clinical detection and monitoring of Alzheimer’s disease. Neurobiol Aging.

[21] Verberk IMW, Thijssen E, Koelewijn J, Mauroo K, Vanbrabant J, de Wilde A, Zwan MD, Verfaillie SCJ, Ossenkoppele R, Barkhof F, van Berckel BNM, Scheltens P, van der Flier WM, Stoops E, Vanderstichele HM, Teunissen CE (2020). Combination of plasma amyloid beta(1-42/1-40) and glial fibrillary acidic protein strongly associates with cerebral amyloid pathology. Alzheimers Res Ther.

[22] Dage JL, Wennberg AMV, Airey DC, Hagen CE, Knopman DS, Machulda MM, Roberts RO, Jack CR, Petersen RC, Mielke MM (2016). Levels of tau protein in plasma are associated with neurodegeneration and cognitive function in a population-based elderly cohort. Alzheimers Dement.

[23] Ding D, Zhao Q, Guo Q, Meng H, Wang B, Yu P, Luo J, Zhou Y, Yu L, Zheng L, Chu S, Mortimer JA, Borenstein AR, Hong Z (2014). The Shanghai Aging Study: study design, baseline characteristics, and prevalence of dementia. Neuroepidemiology..

[24] Luo J, Zhu G, Zhao Q, Guo Q, Meng H, Hong Z, Ding D (2013). Prevalence and risk factors of poor sleep quality among Chinese elderly in an urban community: results from the Shanghai aging study. PLoS One.

[25] Mahoney FI, Barthel DW (1965). Functional evaluation: the Barthel index. Md State Med J.

[26] Lawton MP, Brody EM (1969). Assessment of older people: self-maintaining and instrumental activities of daily living. Gerontologist..

[27] Smirnov DA, Morley M, Shin E, Spielman RS, Cheung VG (2009). Genetic analysis of radiation-induced changes in human gene expression. Nature..

[28] Zhang MY, Katzman R, Salmon D, Jin H, Cai GJ, Wang ZY, Qu G, Grant I, Yu E, Levy P, Klauber MR, Liu WT (1990). The prevalence of dementia and Alzheimer’s disease in Shanghai, China: impact of age, gender, and education. Ann Neurol.

[29] Chen KL, Xu Y, Chu AQ, Ding D, Liang XN, Nasreddine ZS, Dong Q, Hong Z, Zhao QH, Guo QH (2016). Validation of the Chinese version of montreal cognitive assessment basic for screening mild cognitive impairment. J Am Geriatr Soc.

[30] Huang L, Chen KL, Lin BY, Tang L, Zhao QH, Lv YR, Guo QH (2018). Chinese version of Montreal Cognitive Assessment Basic for discrimination among different severities of Alzheimer’s disease. Neuropsychiatr Dis Treat.

[31] Huang YY, Qian SX, Guan QB, Chen KL, Zhao QH, Lu JH, et al. Comparative study of two Chinese versions of Montreal Cognitive Assessment for Screening of Mild Cognitive Impairment. Appl Neuropsychol Adult. 2021;28(1):88–93. 10.1080/23279095.2019.1602530. 10.1080/23279095.2019.1602530 31014115

[32] Ding D, Zhao Q, Guo Q, Meng H, Wang B, Luo J, Mortimer JA, Borenstein AR, Hong Z (2015). Prevalence of mild cognitive impairment in an urban community in China: a cross-sectional analysis of the Shanghai Aging Study. Alzheimers Dement.

[33] Zhao Q, Lv Y, Zhou Y, Hong Z, Guo Q (2012). Short-term delayed recall of auditory verbal learning test is equivalent to long-term delayed recall for identifying amnestic mild cognitive impairment. PLoS One.

[34] Guo Q, Zhao Q, Chen M, Ding D, Hong Z (2009). A comparison study of mild cognitive impairment with 3 memory tests among Chinese individuals. Alzheimer Dis Assoc Disord.

[35] Zhou B, Zhao Q, Kojima S, Ding D, Higashide S, Nagai Y, Guo Q, Kagimura T, Fukushima M, Hong Z (2019). One-year outcome of Shanghai mild cognitive impairment cohort study. Curr Alzheimer Res.

[36] Weixiong S, Chuanzhen LV, Yimin S, Lv CZ, Guo QH (2006). Boston naming test in Chinese elderly: patient with mild cognitive impairment and Alzheimer’s dementia. Chin Ment Health J.

[37] Lu J, Guo Q, Hong Z, Shi W, Lu C (2006). Trail making test used by Chinese elderly patients with mild cognitive impairment and mild Alzheimer’s dementia. Chinese J Clin Psychol.

[38] Nasreddine ZS, Phillips NA, Bédirian V, Charbonneau S, Whitehead V, Collin I (2005). The Montreal Cognitive Assessment, MoCA: a brief screening tool for mild cognitive impairment. J Am Geriatr Soc.

[39] Morris JC (1993). The Clinical Dementia Rating (CDR): current version and scoring rules. Neurology..

[40] Lim WS, Chong MS, Sahadevan S (2007). Utility of the clinical dementia rating in Asian populations. Clin Med Res.

[41] American Psychiatric Association: diagnostic and statistical manual of mental disorders, ed 4. Washington, American Psychiatric Association, 1994, p. 143–147.

[42] McKhann G, Drachman D, Folstein M, Katzman R, Price D, Stadlan EM (1984). Clinical diagnosis of Alzheimer’s disease: report of the NINCDS-ADRDA Work Group under the auspices of Department of Health and Human Services Task Force on Alzheimer’s Disease. Neurology..

[43] Petersen RC (2004). Mild cognitive impairment as a diagnostic entity. J Intern Med.

[44] Petersen RC (2011). Clinical practice. Mild cognitive impairment. New Engl J Med.

[45] Petersen RC, Thomas RG, Grundman M, Bennett D, Doody R, Ferris S, Galasko D, Jin S, Kaye J, Levey A, Pfeiffer E, Sano M, van Dyck CH, Thal LJ (2005). Vitamin E and donepezil for the treatment of mild cognitive impairment. New Engl J Med.

[46] Halawa OA, Gatchel JR, Amariglio RE, Rentz DM, Sperling RA, Johnson KA (2019). Alzheimer’s disease neuroimaging initiative. Inferior and medial temporal tau and cortical amyloid are associated with daily functional impairment in Alzheimer’s disease. Alzheimers Res Ther.

[47] IBM Corp. Released 2017. IBM SPSS Statistics forWindows, Version 25.0. Armonk, NY: IBM Corp.

[48] R Core Team (2020). R: A language and environmentfor statistical computing.

[49] Gaetani L, Blennow K, Calabresi P, Di Filippo M, Parnetti L, Zetterberg H (2019). Neurofilament light chain as a biomarker in neurological disorders. J Neurol Neurosurg Psychiatry.

[50] Villemagne VL, Burnham S, Bourgeat P, Brown B, Ellis KA, Salvado O, Szoeke C, Macaulay SL, Martins R, Maruff P, Ames D, Rowe CC, Masters CL, Australian Imaging Biomarkers and Lifestyle (AIBL) Research Group (2013). Amyloid β deposition, neurodegeneration, and cognitive decline in sporadic Alzheimer’s disease: a prospective cohort study. Lancet Neurol.

[51] Haldenwanger A, Eling P, Kastrup A, Hildebrandt H (2010). Correlation between cognitive impairment and CSF biomarkers in amnesic MCI, non-amnesic MCI, and Alzheimer’s disease. J Alzheimers Dis.

[52] Ovod V, Ramsey KN, Mawuenyega KG, Bollinger JG, Hicks T, Schneider T, Sullivan M, Paumier K, Holtzman DM, Morris JC, Benzinger T, Fagan AM, Patterson BW, Bateman RJ (2017). Amyloid beta concentrations and stable isotope labeling kinetics of human plasma specific to central nervous system amyloidosis. Alzheimers Dement.

[53] Busche MA, Hyman BT (2020). Synergy between amyloid-beta and tau in Alzheimer’s disease. Nat Neurosci.

[54] Weuve J, Proust-Lima C, Power MC, Gross AL, Hofer SM, Thiébaut R, Chêne G, Glymour MM, Dufouil C, MELODEM Initiative (2015). Guidelines for reporting methodological challenges and evaluating potential bias in dementia research. Alzheimers Dement.

[55] Scharfen J, Jansen K, Holling H (2018). Retest effects in working memory capacity tests: a meta-analysis. Psychon Bull Rev.

[56] De Anna F, Attali E, Freynet L, Foubert L, Laurent A, Dubois B (2008). Intrusions in story recall: when over-learned information interferes with episodic memory recall. Evidence from Alzheimer’s disease. Cortex..

[57] Jack CR, Bennett DA, Blennow K, Carrillo MC, Dunn B, Haeberlein SB, Holtzman DM, Jagust W, Jessen F, Karlawish J, Liu E, Molinuevo JL, Montine T, Phelps C, Rankin KP, Rowe CC, Scheltens P, Siemers E, Snyder HM, Sperling R, Elliott C, Masliah E, Ryan L, Silverberg N (2018). NIA-AA Research Framework: toward a biological definition of Alzheimer’s disease. Alzheimers Dement.

