# Supplementary material for: Plasma biomarker profiles and the correlation with cognitive function across the clinical spectrum of Alzheimer’s disease
Source: Alzheimers Res Ther. 2021 Jul 5;13:123. doi: 10.1186/s13195-021-00864-x (PMC8259165; doi:10.1186/s13195-021-00864-x)
Supplement: Supplementary file 1 — Additional file 1: Supplementary Table 1. Domain-specific cognition extracted from neuropsychological tests in the current study. [file 13195_2021_864_MOESM1_ESM.docx]

Supplementary Table 1. Domain-specific cognition extracted from neuropsychological tests in the current study.

| Global cognition | Memory | Attention | Visuospatial function | Language | Executive function |
| --- | --- | --- | --- | --- | --- |
| MMSE | 'delayed recall'  in MMSE | 'registration'  in MMSE | 'overlapping imaging'  in MoCA-B | 'naming'  in MMSE | 'trail making' & 'similarity'  in MoCA-B |
|  |  |  |  |  |  |
|  | 'delayed recall'  in MoCA-B | 'attention'  in MoCA-B | 'copy'  in Rey-Osterrieth Complex Figure | 'naming'  in MoCA-B | 'part B completion time'*  in Trail Making Test (TMT) |
|  |  |  |  |  |  |
|  | 'delayed recall'  in Auditory Verbal Learning Test | 'inhibitory control'  in Conflicting Instructions Task (Go/No-Go Task) | 'copy'  in Stick Test | Boston Naming Test |  |
|  |  |  |  |  |  |
|  |  | Symbol Digit Modalities Test |  | 'objects naming'  in Modified Common Objects Sorting |  |

Note: The score of each test was transformed into percentage of correct answer (%) in all participants [1].

*The performance time values of TMT were divided into three categories to calculate the accuracy according to previously published normative data [2]. The TMT time more than mean + 1.5 standard deviation (SD) was regarded as poor executive function and was assigned 0 score. The TMT time less than mean – 1.5 SD was regarded as good executive function and was assigned 2 scores. Other participants were assigned 1 score. Then, the new TMT scores were also converted to accuracy and *Z*-scores.

Abbreviations: MMSE, mini-mental state examination; MoCA-B, Montreal Cognitive Assessment-Basic; TMT, Trail Making Test.

References:

1. Nathan PJ, Lim YY, Abbott R, Galluzzi S, Marizzoni M, Babiloni C, et al. Association between CSF biomarkers, hippocampal volume and cognitive function in patients with amnestic mild cognitive impairment (MCI). Neurobiol Aging. 2017;53:1-10.
2. Ding D, Zhao Q, Guo Q, Meng H, Wang B, Luo J, et al. Prevalence of mild cognitive impairment in an urban community in China: a cross-sectional analysis of the Shanghai Aging Study. Alzheimers Dement. 2015;11(3):300-9 e2.
3. Nasreddine ZS, Phillips NA, Bédirian V, Charbonneau S, Whitehead V, Collin I, Cummings JL, Chertkow H. The Montreal Cognitive Assessment (MoCA): A Brief Screening Tool For Mild Cognitive Impairment. Journal of the American Geriatrics Society 53:695-699, 2005.
4. Chen KL, Xu Y, Chu AQ, Ding D, Liang XN, Nasreddine ZS, Dong Q, Hong Z, Zhao QH, Guo QH. Validation of the Chinese Version of Montreal Cognitive Assessment Basic for Screening Mild Cognitive Impairment. J Am Geriatr Soc. 2016 Dec;64(12):e285-e290. doi: 10.1111/jgs.14530. Epub 2016 Nov 7. PMID: 27996103.
5. Guo Q, Zhao Q, Chen M, Ding D, Hong Z. A comparison study of mild cognitive impairment with 3 memory tests among Chinese individuals. Alzheimer Dis Assoc Disord. 2009 Jul-Sep;23(3):253-9. doi: 10.1097/WAD.0b013e3181999e92. PMID: 19812468.
6. Zhao Q, Guo Q, Li F, Zhou Y, Wang B, Hong Z. The Shape Trail Test: application of a new variant of the Trail making test. PLoS One. 2013;8(2):e57333. doi: 10.1371/journal.pone.0057333. Epub 2013 Feb 20. PMID: 23437370; PMCID: PMC3577727.
7. Zhou B, Zhao Q, Kojima S, Ding D, Higashide S, Nagai Y, Guo Q, Kagimura T, Fukushima M, Hong Z. One-year Outcome of Shanghai Mild Cognitive Impairment Cohort Study. Curr Alzheimer Res. 2019;16(2):156-165. doi: 10.2174/1567205016666181128151144. PMID: 30484408.
