# Supplementary material for: Plasma biomarker profiles and the correlation with cognitive function across the clinical spectrum of Alzheimer’s disease
Source: Alzheimers Res Ther. 2021 Jul 5;13:123. doi: 10.1186/s13195-021-00864-x (PMC8259165; doi:10.1186/s13195-021-00864-x)
Supplement: Supplementary file 2 — Additional file 2: Supplementary Table 2. Correlations between plasma biomarkers and global & domain-specific cognition in participants with different AD stages [file 13195_2021_864_MOESM2_ESM.docx]

| Supplementary Table 2. Correlations between plasma biomarkers and global & domain-specific cognition in participants with different AD stages | | | | | | | | | | | | | | | | | | | | | | | | | | |
| --- | --- | --- | --- | --- | --- | --- | --- | --- | --- | --- | --- | --- | --- | --- | --- | --- | --- | --- | --- | --- | --- | --- | --- | --- | --- | --- |
|  | Global (MMSE) | | | | | | | |  | Memory | | | | | | | |  | Attention | | | | | | | |
|  | CDR = 0 | |  | CDR = 0.5 | |  | CDR ≥ 1 | |  | CDR = 0 | |  | CDR = 0.5 | |  | CDR ≥ 1 | |  | CDR = 0 | |  | CDR = 0.5 | |  | CDR ≥ 1 | |
| Biomarkers | *r* | *P* |  | *r* | *P* |  | *r* | *P* |  | *r* | *P* |  | *r* | *P* |  | *r* | *P* |  | *r* | *P* |  | *r* | *P* |  | *r* | *P* |
| Plasma Aβ_1-40_ | -0.125 | 0.162 |  | 0.070 | 0.394 |  | -0.029 | 0.715 |  | -0.053 | 0.565 |  | 0.014 | 0.866 |  | 0.093 | 0.272 |  | -0.056 | 0.536 |  | -0.065 | 0.446 |  | -0.062 | 0.468 |
| Plasma Aβ_1-42_ | -0.092 | 0.306 |  | -0.091 | 0.268 |  | 0.073 | 0.351 |  | 0.008 | 0.934 |  | 0.154 | 0.068 |  | 0.202 | 0.017 |  | -0.139 | 0.125 |  | -0.016 | 0.850 |  | 0.034 | 0.687 |
| Plasma Aβ_1-42_/Aβ_1-40_ | -0.002 | 0.985 |  | -0.163 | 0.048 |  | 0.159 | 0.041 |  | 0.025 | 0.782 |  | 0.160 | 0.057 |  | 0.124 | 0.144 |  | -0.115 | 0.205 |  | 0.003 | 0.968 |  | 0.184 | 0.029 |
| Plasma t-tau | -0.046 | 0.610 |  | -0.136 | 0.097 |  | -0.134 | 0.087 |  | -0.154 | 0.089 |  | -0.177 | 0.036 |  | -0.060 | 0.482 |  | -0.107 | 0.237 |  | -0.090 | 0.287 |  | -0.042 | 0.619 |
| Plasma NfL | -0.160 | 0.073 |  | -0.069 | 0.401 |  | -0.131 | 0.097 |  | 0.069 | 0.450 |  | -0.095 | 0.262 |  | -0.156 | 0.070 |  | -0.089 | 0.324 |  | -0.032 | 0.710 |  | 0.030 | 0.731 |
| Plasma p-tau181 | -0.044 | 0.624 |  | -0.134 | 0.106 |  | -0.301 | <0.001* |  | -0.001 | 0.989 |  | -0.214 | 0.011 |  | -0.192 | 0.024 |  | 0.010 | 0.909 |  | -0.020 | 0.818 |  | -0.180 | 0.035 |
|  |  |  |  |  |  |  |  |  |  |  |  |  |  |  |  |  |  |  |  |  |  |  |  |  |  |  |
|  | Visuospatial function | | | | | | | |  | Language | | | | | | | |  | Executive function | | | | | | | |
|  | CDR = 0 | |  | CDR = 0.5 | |  | CDR ≥ 1 | |  | CDR = 0 | |  | CDR = 0.5 | |  | CDR ≥ 1 | |  | CDR = 0 | |  | CDR = 0.5 | |  | CDR ≥ 1 | |
| Biomarkers | *r* | *P* |  | *r* | *P* |  | *r* | *P* |  | *r* | *P* |  | *r* | *P* |  | *r* | *P* |  | *r* | *P* |  | *r* | *P* |  | *r* | *P* |
| Plasma Aβ_1-40_ | -0.099 | 0.275 |  | -0.039 | 0.659 |  | 0.091 | 0.321 |  | -0.164 | 0.070 |  | -0.098 | 0.248 |  | -0.035 | 0.682 |  | -0.008 | 0.931 |  | 0.049 | 0.563 |  | -0.001 | 0.992 |
| Plasma Aβ_1-42_ | -0.002 | 0.986 |  | -0.142 | 0.104 |  | 0.099 | 0.278 |  | -0.102 | 0.261 |  | -0.125 | 0.137 |  | -0.0003 | 0.997 |  | -0.066 | 0.483 |  | -0.119 | 0.163 |  | 0.063 | 0.460 |
| Plasma Aβ_1-42_/Aβ_1-40_ | 0.087 | 0.339 |  | -0.108 | 0.217 |  | 0.040 | 0.661 |  | 0.084 | 0.355 |  | -0.090 | 0.289 |  | 0.043 | 0.613 |  | -0.084 | 0.366 |  | -0.204 | 0.016 |  | 0.086 | 0.315 |
| Plasma t-tau | 0.043 | 0.638 |  | 0.101 | 0.247 |  | -0.100 | 0.277 |  | -0.011 | 0.903 |  | -0.063 | 0.456 |  | -0.025 | 0.773 |  | -0.004 | 0.970 |  | 0.025 | 0.773 |  | -0.032 | 0.708 |
| Plasma NfL | -0.034 | 0.708 |  | -0.181 | 0.037 |  | 0.041 | 0.657 |  | -0.153 | 0.092 |  | 0.035 | 0.675 |  | 0.007 | 0.934 |  | 0.067 | 0.476 |  | 0.131 | 0.124 |  | -0.171 | 0.047 |
| Plasma p-tau181 | 0.084 | 0.358 |  | -0.071 | 0.419 |  | -0.057 | 0.540 |  | -0.041 | 0.650 |  | 0.058 | 0.496 |  | 0.054 | 0.532 |  | 0.001 | 0.993 |  | 0.141 | 0.096 |  | -0.063 | 0.467 |
| Note: The plasma biomarkers concentrations were log transformed. The partial correlation coefficients (*r*) were adjusted for age, gender, and education year. * P < 0.00046 was considered statistically significant after Bonferroni correction for multiple comparisons. CDR, Clinical Dementia Rating Scale; Aβ, amyloid-beta protein; t-tau, total tau; NfL, neurofilament protein light chain; p-tau181, tau phosphorylated at threonine 181. | | | | | | | | | | | | | | | | | | | | | | | | | | |
