# Supplementary material for: Plasma biomarker profiles and the correlation with cognitive function across the clinical spectrum of Alzheimer’s disease
Source: Alzheimers Res Ther. 2021 Jul 5;13:123. doi: 10.1186/s13195-021-00864-x (PMC8259165; doi:10.1186/s13195-021-00864-x)
Supplement: Supplementary file 3 — Additional file 3: Supplementary Table 3. Intrusion of the Memory tests. [file 13195_2021_864_MOESM3_ESM.docx]

Supplementary Table 3. Intrusion of the Memory tests.

|  |  | Clinical diagnosis | | | | | |
| --- | --- | --- | --- | --- | --- | --- | --- |
|  |  | NC | aMCI-s | aMCI-m | Mild AD | Moderate AD | Severe AD |
|  | Total | CDR = 0 | CDR = 0.5 | CDR = 0.5 | CDR = 1 | CDR = 2 | CDR = 3 |
|  | (N = 451) | (N = 131) | (N = 39) | (N = 113) | (N = 67) | (N = 63) | (N = 38) |
| Intrusion, n (%) | 174 (38.6) | 0 (0) | 22 (56.4) | 98 (86.7) | 34 (50.7) | 15 (23.8) | 5 (13.2) |

Note: NC, normal cognition; aMCI-s, amnestic mild cognitive impairment-single domain; aMCI-m, amnestic mild cognitive impairment-multiple domains; AD, Alzheimer's disease clinical syndrome; CDR, Clinical Dementia Rating Scale.
