# Supplementary material for: Plasma biomarker profiles and the correlation with cognitive function across the clinical spectrum of Alzheimer’s disease
Source: Alzheimers Res Ther. 2021 Jul 5;13:123. doi: 10.1186/s13195-021-00864-x (PMC8259165; doi:10.1186/s13195-021-00864-x)
Supplement: Supplementary file 4 — Additional file 4: Supplementary Table 4. Correlations between plasma biomarkers and cognitive domains in participants that were able to accomplish all tests (N = 374). [file 13195_2021_864_MOESM4_ESM.docx]

Supplementary Table 4. Correlations between plasma biomarkers and cognitive domains in participants that were able to accomplish all tests (N = 374).

|  | Global | | Memory | | Attention | | Visuospatial function | | Language | | Executive function | |
| --- | --- | --- | --- | --- | --- | --- | --- | --- | --- | --- | --- | --- |
|  | *r* | *P* | *r* | *P* | *r* | *P* | *r* | *P* | *r* | *P* | *r* | *P* |
| Plasma Aβ40 | 0.074 | 0.157 | 0.119 | 0.022 | 0.085 | 0.105 | 0.083 | 0.111 | 0.009 | 0.860 | 0.077 | 0.142 |
| Plasma Aβ42 | 0.136 | 0.0088 | 0.243 | **<0.0001** | 0.149 | 0.004 | 0.100 | 0.056 | 0.029 | 0.576 | 0.024 | 0.641 |
| Plasma Aβ42/Aβ40 | 0.117 | 0.025 | 0.183 | **0.0004** | 0.114 | 0.029 | 0.062 | 0.237 | 0.016 | 0.763 | -0.062 | 0.238 |
| Plasma t-tau | -0.175 | **0.0007** | -0.129 | 0.013 | -0.111 | 0.033 | -0.035 | 0.498 | -0.047 | 0.373 | 0.007 | 0.899 |
| Plasma NfL | -0.243 | **<0.0001** | -0.259 | **<0.0001** | -0.224 | **<0.0001** | -0.235 | **<0.0001** | -0.107 | 0.040 | -0.067 | 0.201 |
| Plasma p-tau181 | -0.525 | **<0.0001** | -0.468 | **<0.0001** | -0.449 | **<0.0001** | -0.358 | **<0.0001** | -0.163 | 0.0018 | -0.129 | 0.013 |

Note: The plasma biomarkers concentrations were log transformed. The partial correlation coefficients (r) were adjusted for age, gender, and education year. Bold *P*s (< 0.0014) were considered statistically significant after using multiple comparisons by Bonferroni correction. Aβ, amyloid-beta protein; t-tau, total tau; NfL, neurofilament protein light chain; p-tau181, tau phosphorylated at threonine 181.
